# On the Use of Formalin as a Preservative Fluid

**Published:** 1896-05

**Authors:** J. T. Duncan

**Affiliations:** Professor of Anatomy, Ontario Veterinary College, Toronto


### ON THE USE OF FORMALIN AS A PRESERVATIVE FLUID.

By J. T. DUNCAN, M.D.,
PROFESSOR OF ANATOMY, ONTARIO VETERINARY COLLEGE, TORONTO.

FORMALIN is a name given to a solution of formic aldehyde in water of 40 per cent. strength. It has come considerably into use in the last year. The first notice of it which came under my observation was contained in the *British Medical Journal*, (1894, vol. ii., page 1429), when the following words were used: "A 10 per cent. solution of this was best suited for hardening purposes. If, after removal, an eye was immersed in this for twenty-four hours it became perfectly hard, and could be cut easily; the eye retained its natural, fresh appearance: the cornea and lens were transparent; the iris was normal; blood and pus were unchanged in appearance; the vitreous was unaltered, and the retina and choroid remained *in situ*. There was no need to freeze the eye in order to cut it, as was necessary after hardening in Müller's fluid." Another notice in the same journal stated that a 20 per cent. solution hardened a brain perfectly in two days, while for gradual hardening, or as a preservative agent for anatomical and pathological specimens, a 1 per cent. solution was sufficient

In the museum of the Ontario Veterinary College we had up to the above-mentioned period used alcohol (methylated) as a preservative fluid almost exclusively. But it has several disadvantages. It shrinks the tissues considerably, in many cases decolorizes them, while for certain purposes it is quite unsuitable. In the hope of finding a better agent, it was determined to examine the possibilities of formalin.

The first experiment was with the eye of a horse, which I desired to harden for demonstration purposes. A 10 per cent. solution was used, the fresh eyes remaining in it for two days. In sectioning these specimens the result bore out the statement quoted from the journal. But the vitreous was not hardened and the aqueous humor was quite fluid, escaping freely from its chambers.[1]

Some of our transverse sections of the thorax require receptacles of considerable size. These are not hermetically sealed, but simply covered by a large glass laid on the flanged top. Receptacles covered in such a manner would allow a large evaporation of alcohol or of any fluid used in this way. Into one such tin (which was painted and prepared with plaster-of-Paris in exactly the same way as those containing alcohol) we placed transverse thoracic sections three inches thick and covered them with a 1½ per cent. solution of formalin. This was done last spring (1895), and the ensuing summer was a very hot one. It was, therefore, a good test of the capabilities of the new fluid.

Before giving the result of the experiment I may state the

[1] Further experiments show that formalin will not solidify the aqueous. I have just cut an eye in which 100 per cent. (pure formalin) was used. The aqueous was quite fluid. The vitreous, while perfectly clear and jelly-like, remained *in situ*, although the section was turned with the cut side down and shaken. This would go far to prove that the vitreous is held in position by delicate processes of the hyaloid membrane passing through it; although this is denied by many authorities.

two principal objections which some writers prefer to the use of formalin. First, it is said to be necessary constantly to add formalin to the solution, otherwise it becomes too weak to preserve the immersed material. (This objection would only apply, I presume, to jars not hermetically closed.) The second objection is, that unless the specimens are completely covered the exposed parts will become mouldy.

In the experiment here, of which I am speaking, no formalin has been added since the solution was first made. The results may be thus stated:

1. The solution has evaporated to some extent, so that portions of the sections are above water.

2. No mould has yet appeared. (I am purposely leaving the sections as they are. Some weeks ago I fancied I saw a speck of mould, but as it has not increased in size I do not think it is of that nature.)

3. No discoloration has taken place; the sections look almost as fresh as when cut.

4. The lung-tissue is sufficiently firm and well preserved, yet a very little shrinking of it has taken place.

5. The fluid itself remains beautifully clear.

6. Comparing the tins standing side by side with this, the decoloration of some of the tissues by alcohol is very marked, while the evaporation from those tins has gone to as great or a still greater extent than from the one containing formalin.

For hardening brains for demonstration purposes we find a 10 per cent. solution the best agent yet tried. They will be in excellent order for cutting in two to three weeks. It is not necessary to inject the brain, but merely place it in the fluid.

In regard to the comparative cost of alcohol and formalin, the formalin solution is very much the cheaper of the two.

The results of the various tests being, so far, very favorable, we have made arrangements for its further use during the present season.

---

A two-year-old colt was gelded April 29, 1895, near Toledo Ohio. Two days later he was turned out with a mare. April 1st last the mare dropped a horse foal.

